# Long-Term Results of Stereotactic Radiotherapy in Patients with at Least 10 Brain Metastases at Diagnosis

**DOI:** 10.3390/cancers16091742

**Published:** 2024-04-29

**Authors:** Rémy Kinj, Andreas Felix Hottinger, Till Tobias Böhlen, Mahmut Ozsahin, Véronique Vallet, Vincent Dunet, Hasna Bouchaab, Solange Peters, Constantin Tuleasca, Jean Bourhis, Luis Schiappacasse

**Affiliations:** 1 Department of Radiation Oncology, Lausanne University Hospital and University of Lausanne, CHUV, Rue du Bugnon 46, CH-1011 Lausanne, Switzerland; 2 Lundin Family Brain Tumor Centre, Departments of Oncology & Clinical Neurosciences, Hospital and University of Lausanne, CH-1011 Lausanne, Switzerland; 3 Departments of Medical Oncology & Clinical Neurosciences, Lausanne University Hospital and University of Lausanne, CH-1011 Lausanne, Switzerland; 4 Institute of Radiation Physics, Lausanne University Hospital and University of Lausanne, CH-1011 Lausanne, Switzerland; 5 Departement of Medical Radiology, Lausanne University Hospital and University of Lausanne, CH-1011 Lausanne, Switzerland; 6 Departments of Medical Oncology, Lausanne University Hospital and University of Lausanne, CH-1011 Lausanne, Switzerland; 7 Department of Clinical Neurosciences, Neurosurgery Service and Gamma Knife Center, Lausanne University Hospital, University of Lausanne, CH-1011 Lausanne, Switzerland

**Keywords:** stereotactic radiotherapy, brain metastases, SRS

## Abstract

**Simple Summary:**

Cancer patients presenting with at least 10 lesions at brain metastasis (BM) diagnosis usually receive palliative whole-brain radiotherapy (WBRT). Our institutional BM tumor board favors the use of stereotactic radiotherapy (SRT) as much as possible, even in this population. This approach leads to an excellent local control rate of BM, with a low toxicity and radio-necrosis rate. Close MRI surveillance every 2 months is necessary for early detection in cases of new BMs, and repeated brain SRT is feasible and safe. Less than half of the patients will require a subsequent brain SRT. Moreover, at one year, less than a quarter of patients require a salvage WBRT or die from a neurological progression (strategy success rate). Overall survival data seem favorable compared to historical outcomes after WBRT in this population.

**Abstract:**

Purpose: to evaluate an SRT approach in patients with at least 10 lesions at the time of BM initial diagnosis. Methods: This is a monocentric prospective cohort of patients treated by SRT, followed by a brain MRI every two months. Subsequent SRT could be delivered in cases of new BMs during follow-up. The main endpoints were local control rate (LCR), overall survival (OS), and strategy success rate (SSR). Acute and late toxicity were evaluated. Results: Seventy patients were included from October 2014 to January 2019, and the most frequent primary diagnosis was non-small-cell lung cancer (N = 36, 51.4%). A total of 1174 BMs were treated at first treatment, corresponding to a median number of 14 BMs per patient. Most of the patients (N = 51, 72.6%) received a single fraction of 20–24 Gy. At 1 year, OS was 62.3%, with a median OS of 19.2 months, and SSR was 77.8%. A cumulative number of 1537 BM were treated over time, corresponding to a median cumulative number of 16 BM per patient. At 1-year, the LCR was 97.3%, with a cumulative incidence of radio-necrosis of 2.1% per lesion. Three patients (4.3%) presented Grade 2 toxicity, and there was no Grade ≥ 3 toxicity. The number of treated BMs and the treatment volume did not influence OS or SSR (*p* > 0.05). Conclusions: SRT was highly efficient in controlling the BM, with minimal side effects. In this setting, an SRT treatment should be proposed even in patients with ≥10 BMs at diagnosis.

## 1. Introduction

Brain metastases (BMs) are frequent in patients with cancer and have a direct impact on life expectancy and quality of life. The prognosis of patients with BMs depends on the histological type, number of BMs, extracranial tumor burden, patient performance status, and availability of effective treatments [1]. The risk of developing BMs varies by tumor type, and melanoma (40–60%), lung cancer (20–45%), and breast cancer (5–30%) have the highest risk [2,3,4].

In cases of limited brain metastases, extension stereotactic radiotherapy (SRT) or surgery are the preferred options. In cases of extensive brain metastases, different treatment modalities, such as systemic treatment, whole brain radiotherapy (WBRT), or SRT in patients with low overall tumor volume and/or radioresistant tumors (such as melanoma) can be discussed [5]. In addition to WBRT, the management in cases of ≥10 BMs relies on a multidisciplinary approach combining systemic treatments effective for BM (i.e., EGFR-tyrosine-kinase inhibitors, immunotherapy, ALK inhibitors, etc.), stereotactic radiotherapy (SRT), and, rarely, neurosurgery (i.e., symptomatic mass, selected microsurgical resection, etc.) [6,7,8]. Moreover, patients with multiple BMs present a possible intra-tumoral heterogeneity and treatment resistance, as well as the risk of recurrence despite aggressive local therapies [9].

WBRT is still considered as one of the favorite options for patients with ≥10 BMs. This treatment is usually delivered in 10 fractions, irradiating healthy brain tissue and BMs at the same dose of 30 Gy [10]. The doses delivered during WBRT are palliative and do not permit a permanent control of the irradiated metastases [10]. The outcome after WBRT is poor and, in the absence of further treatments, most patients die from BM progression [11]. Moreover, WBRT carries multiple side effects, both acute and delayed. Headache, nausea, asthenia, and alopecia are common during the first 3 months after irradiation. Disabling cognitive and memory disorders appear at a later stage, for surviving patients [10].

Stereotactic radiotherapy (SRT) allows scientists to deliver a high curative irradiation dose to each BM, resulting in a high rate of local control [12]. Due to SRT’s high accuracy, a minimal volume of healthy brain parenchyma is irradiated, thus resulting in very few side effects [13,14]. In the absence of prophylaxis with an SRT approach, patients should be given repeated brain imaging and may require the repetition of brain SRT (mostly in cases of new BMs). The use of SRT is commonly accepted when patients present up to five BMs. Under certain conditions (low cumulative tumor burden), brain SRT can be discussed for up to 10 metastases [15,16,17,18]. However, the conditions for using stereotactic irradiation beyond 10 BMs needs to be explored and specified, and its use in these conditions remains limited to a few expert centers [16,19,20].

Our institutional brain metastases tumor board has adopted an approach that favors, as much as possible, the use of SRT in patients even with ≥10 BMs at BM diagnosis. The clinical criteria to benefit from SRT are not restrictive and reach up to clinical and technical limits. We defined the limits as follows: patients must be free from grade ≥ 3 neurological symptoms, must present a quantifiable and treatable number of BMs (based on clinical experience: <40 BM, except for radio-resistant histology, such as melanoma or renal cell carcinoma up to 70 BM, all lesion diameter < 4 cm), and must show no evidence of leptomeningeal dissemination. Beyond those limits, palliative WBRT is considered.

Our study aimed to analyze clinical, radiological, and dosimetric data of patients who were treated with SRT on at least 10 lesions at BM initial diagnosis. The objective was to analyze the long-term outcome of these patients and to determine the efficacy and toxicity of these treatments, as well as potential prognostic factors.

## 2. Materials and Methods

This monocentric prospective cohort study included patients treated with at least 10 lesions at initial BM diagnosis from October 2014 to January 2019. All SRT indications were discussed, approved, and registered by a weekly institutional brain metastases tumor board (neurosurgeons, neuroradiologists, radiation oncologists, oncologists, and neurologists). The eligibility criteria were as follows: ≥10 BMs at first BM diagnosis, first SRT treatment. Exclusion criteria were as follows: history of WBRT, history of any brain radiotherapy, less than 10 brain metastases. Conditions to benefit from an SRT approach were detailed above.

Clinical, radiological, and dosimetrical data were reported. Clinical data, such as the patient’s age, sex, ECOG performance status score, the origin of the primary and its extra-cranial status (controlled/uncontrolled), RTOG-RPA classification, Ds-GPA classification, and concomitant anti-tumor medication, were collected [21,22,23]. Toxicity was reported according to the Common Terminology Criteria for Adverse Events (CTCAE) v 4.0 [24]. The study was approved by the cantonal ethics committee (CER-VD, Project ID 2023-00580).

The fractionation schedule (dose and number of fractions), the gross tumor volume (GTV), the planning treatment volume (PTV), the dose received by the brain, (i.e., the mean brain dose (MBD), the volume of the brain receiving 12 Gy outside the PTV (Brain-PTV-V12)), and cumulative dosimetry in the event of repeated courses of SRT were recorded. Radiological data, including outcomes after SRT and the occurrence of radio-necrosis, were collected prospectively. Clinical and radiological data were extracted from the institutional clinical software Soarian^®^ (Cerner, North Kansas City, MO, USA) and Archimède^®^ (CHUV) software; dosimetric data were recorded from the RayStation^®^ (RaySearch Laboratories, Stockholm, Sweden) and VelocityTM (Varian Medical Systems, Palo Alto, CA, USA) radiotherapy software v4.1.

Prescribed radiation doses depended on lesion size; for lesions less than 20 mm in the longer axis, a single dose of 20 or 24 Gy (depending on the histology of the primary) at isodose 72% was delivered; for lesions larger than 30 mm in the longer axis, a fractionated treatment was delivered (35 Gy in 5 fractions at isodose 80%). Between 20 and 30 mm, a case-by-case decision was made to determine the optimal fractionation. The GTV to PTV margin was 1 mm. Dose summations were performed in order to assess cumulative doses to organs at risk. Radiotherapy was delivered in strict conditions: a maximal delay of 7 days between planning MRI and SRT had to be respected. Irradiation was delivered using the stereotactic Cyberknife© device (Accuray, Madison, WI, USA), which offers non-isocentric treatments. Patients benefited from a clinical and a radiological (brain MRI) follow-up every two months. The MRI protocol included at least the following sequences: 3D-T1 isotropic at 1 mm resolution before and after injection of a double or triple dose of gadolinium contrast agent, 2D-T2 transverse spin echo (T2SE), transverse FLAIR (fluid-attenuated inversion recovery), and diffusion sequences [25]. Susceptibility (SWI) or T2 gradient echo (T2GE), perfusion, and spectroscopy sequences may be added in addition. On follow-up imaging, the response of each treated lesion was assessed as follows: complete response was considered when the lesion disappeared, partial response was considered when the lesion’s longest diameter decreased ≥30%, stable disease was considered when the lesion’s longest diameter decreased <30% or increased <20% with a gado-T1/T2 match, recurrence/progression of the disease was considered when the lesion’s longest diameter increased ≥20% with a gado-T1/T2 match, and radio-necrosis was considered when the lesion’s longest diameter increased either < or ≥20% with a gado-T1/T2 mismatch [26]. New parenchymal, meningeal, and bony lesions were additionally looked for. In case of the occurrence/suspicion of new BM or of recurrence/radio-necrosis, cases were rediscussed in our multidisciplinary BM tumor board [27]. Patients could then benefit from repeated brain SRT in cases of confirmed new BMs.

Time-to-event outcomes were calculated from the last day of initial brain SRT. We defined strategy success rate (SRR) as a composite endpoint that corresponds to the proportion of patients not requiring WBRT and not dead from a neurological progression. Freedom from WBRT represents the proportion of patients not requiring WBRT at any time. Indeed, patients could necessitate a salvage WBRT in case of brain relapse that did not fulfill the eligibility criteria for a subsequent SRT. The local control rate was defined through the absence of any local failure of the irradiated BM. Statistical analyses were performed using the JMP^®^ 17 software (SAS Institute, Cary, NC, USA). Survival data were analyzed by using the Kaplan–Meier method, and comparisons between groups were conducted by using the Log-rank test for censored data [28,29]. Statistical significance was achieved if the *p*-value was <0.05. Confidence intervals (CI) were computed from standard errors. For analysis of quantitative variables, cut-offs were based on the variable’s median value. Log-rank analysis was performed in order to identify potential factors correlated with outcome. If the *p*-value was ≤0.10 in the univariate analysis, the variable was tested in a multivariate analysis through the Cox regression test [30].

## 3. Results

**Figure 1:**
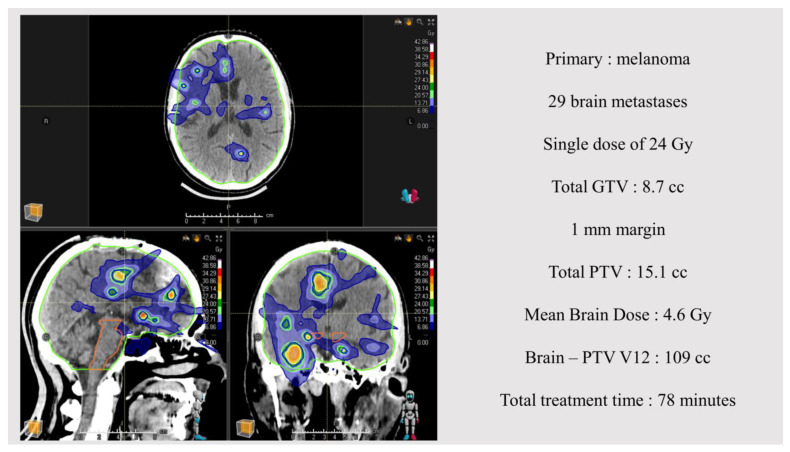
Stereotactic radiotherapy treatment for brain metastases synchronous with a melanoma diagnosis.

Over 879 patients presented to the BM tumor board during the timelapse; 70 fulfilled the inclusion criteria and were included. The median age of patients was 64.4 (min–max range: 24.8–86.1) years and 52.8% (N = 37) were women. The most frequent primary type was non-small-cell lung cancer (NSCLC) (N = 36, 51.4%) mostly adenocarcinoma (N = 33, 47.1%), followed by melanoma (N = 12, 17.0%). The most frequent concomitant systemic treatment was immunotherapy alone (PD1-inhibitors, N = 21, 30.0%). Patients were mostly in good general condition (ECOG PS 0–1: N = 56, 80.0%). A total number of 1174 BMs were treated at first diagnosis, corresponding to a median number of 14 BMs per patient (min–max range: 10–64). A majority of patients (N = 51, 72.6%) benefited from single-dose irradiation of 20 Gy (N = 42) or 24 Gy (N = 9), and the MBD was 3.8 Gy (SD: 1.3–8.9). Other clinical and dosimetric data are presented in Table 1. An illustrative example of an initial brain SRT treatment is presented in Figure 1.

**Figure 2:**
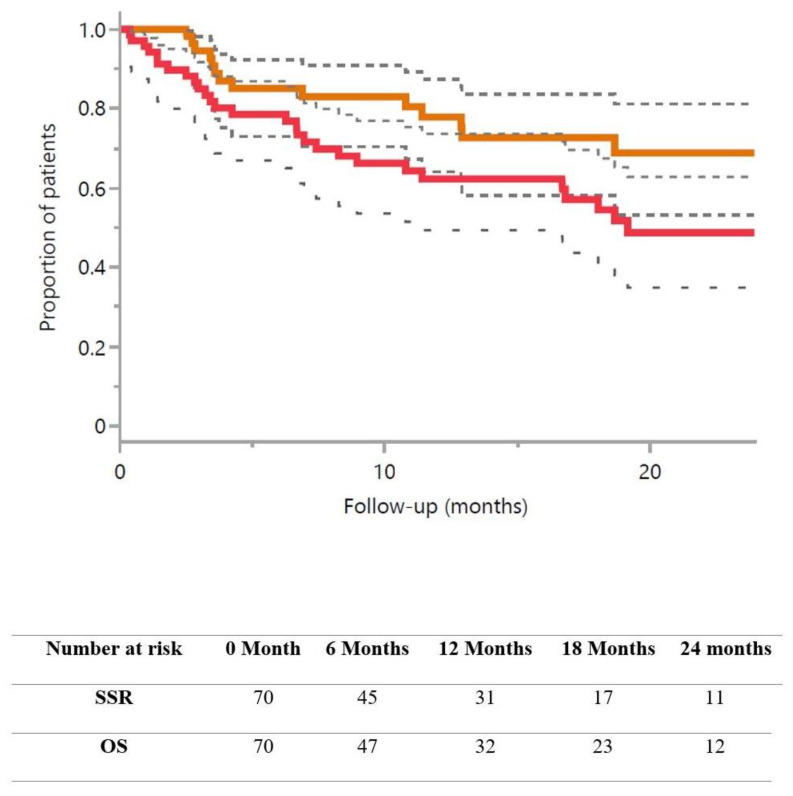
Strategy success rate (brown line), overall survival (red line), and confidence interval (dashed line).

With a median follow up of 18.8 months, the 1 and 2 year OS rates were, respectively, 62.3% (95% CI 50.0–74.6) and 48.7% (95% CI 34.1–63.0) with a median OS of 19.2 months (95% CI 3.6–55.1) and an SSR rate at 1-year of 77.8% (95% CI 66.1–89.5) (Figure 2). Freedom from WBRT at 1 year was 87.8% (95% CI 78.8–96.8), and the median time to a new BM was 4.1 months (95% CI 1.6–22.0) (Table 2).

A majority of patients (77.1%) required only one or two sequences of brain SRT, while nine patients (12.8%) were treated four or more times. A total cumulative number of 1537 BMs were treated overtime, corresponding to a median cumulative number of 16 (10–105) BMs per patient, resulting in a cumulative MBD of 4.9 Gy (min–max range: 1.3–11.0) (Table 2 and Table 3).

Respectively, at 1 and 2 years of follow-up, the local control rates (LCRs) per BM were 97.3% (95% CI 96.4–98.2) and 96.4% (95% CI 95.0–97.8), and the cumulative incidences of radiological radio-necrosis were 2.1% (95% CI 1.0–3.1) and 2.9% (95% CI 1.5–4.3) in a total of 10 individual patients (14.3%). A low proportion of patients (three patients, 4.3%) presented Grade ≥ 2 toxicity; one presented acute Grade 2 post-SRT asthenia, one presented an acute Grade 2 epilepsy crisis, and one presented late Grade 2 radio-necrosis. There was no grade ≥ 3 toxicity. Other characteristics are described in Table 2.

Prognostic factors for OS in univariate analyses revealed the significant influence of sex, ECOG performance status, RPA classification, and origin of the primary (*p* < 0.05). After Cox regression analysis, RPA-3 independently remained associated with a deteriorated OS. Indeed, the relative risk (RR) of death of patients with RPA-3 was significantly higher (RR = 5.5 (2.24–13.2, *p* = 0.0004)) than those with RPA 1–2 (Table 4). We could highlight that the number of BMs and the treatment volume (PTV) did not significantly influence the outcome of patients in terms of OS and SSR (*p* > 0.05).

## 4. Discussion

The choice of our institutional brain metastases tumor board was to adopt an approach that favors, as much as possible, the use of SRT in patients even with ≥10 BMs at BM diagnosis. WBRT indications are restricted to a limited number of patients with advanced cases that exceed the clinical and technical limits that we defined previously. Moreover, a careful imaging follow-up (MRI every 2 months) permitted early detection in cases of a new BM appearance. Our results suggest that an SRT approach in patients with ≥ 10 BMs at BM diagnosis is feasible, with a high efficacy and a low toxicity.

In this population, the choice to deliver a palliative WBRT rather than to use a curative multi-SRT approach is commonly justified by an expected uncontrollable neurological progression and a short life expectancy. However, in our study, the OS outcome was strikingly good, with OS values at 1 and 2 years, respectively, of 62.3% (50.0–74.6) and 48.7% (34.1–63.0), and a median OS of 19.2 months (3.6–55.1). These results compared favorably to the outcome of the JLGK0901 trial that treated patients with SRT for up to 10 BMs and found a median OS of 13.9 months [16]. This is in contrast with Yamamoto et al., who published the outcome of a multi-institutional cohort of patients including patients with more than 10 BM, revealing a median OS of 6.5 months in this subset of patients [19]. The favorable OS results obtained in our study could be explained by several factors, such as clinical characteristics and the implementation of a rigorous post-SRT follow-up.

First, the precited studies included patients with BMs at any time in their BM clinical history, while our study only focused on patients who presented ≥10 BMs at initial BM diagnosis and were naive to any previous BM radiotherapy.

Secondly, most of the patients in the other studies were treated more than a decade ago, in comparison to numerous patients of our cohort who could benefit from modern systemic treatments, such as immunotherapy (30.0%), molecular targeted therapy (15.7%), or chemo-immunotherapy combination (7.1%). These new oncologic treatments have been shown to significantly prolong extracranial disease control and OS of metastatic patients. A third point is that we performed a strict follow-up MRI every two months, permitting the early detection and efficient treatment of any new BM.

Finally, some clinical and technical preconditions were respected in our cohort and could explain the good results that we found. The preconditions to ensure the reproducibility of these results and potentially to routinely broadcast this approach of SRT in cases of ≥10 BMs are as follows: (1) patients should have a quantifiable and treatable number of BM without grade ≥ 3 neurological symptoms and without evidence of leptomeningeal dissemination; (2) a rigorous MRI follow-up every two months is needed, and a multidisciplinary tumor board is necessary to be able to better assess the situation in the case of progression/radio-necrosis doubt; (3) SRT has to be delivered in strict conditions: a cumulative radiotherapy plan must be calculated before treatment validation, a delay of ≤7 days between planning MRI and SRT start must be respected, a very precise SRT device allowing a high gradient of dosage must be employed (allowing optimal PTV margins of ≤1 mm) to limit high doses to surrounding healthy brain tissues and to reduce MBD.

Consistent with other studies, we did not find an impact of the number of BMs in influencing OS [16,19,31,32]. SSR was also not influenced by the number of treated BMs or by the treatment volume (PTV). As expected, the deteriorated general condition of patients (ECOG PS ≥ 2, RPA-3) was associated with a poor prognosis for OS [19].

The most represented primary type was lung. We can note that in a retrospective cohort study of 239 patients, an SRT approach led to an OS improvement compared to a WBRT approach in a population of patients presenting metastatic EGFR-mutated lung cancer with BM (HR = 0.38, 95% CI 0.17–0.84, *p* = 0.017) [33]. Moreover, in another cohort of 121 patients also presenting metastatic EGFR-mutated lung cancer with BM and treated by osimertinib, an upfront SRT treatment of all BMs revealed a better OS outcome compared to osimertinib alone (HR = 0.37, 95% CI 0.16–0.87, *p* = 0.021) [34].

The SSR is a composite endpoint that was used in this study, as it can reflect to what extent the SRT approach was successful in this population. The SSR of 77.8% (66.1–89.5) at 1 year indicates that only 22.2% of patients required either a salvage WBRT or died from a neurological progression. There was no difference in terms of SSR depending on patients’ general condition. The absence of influence of this parameter supports the appropriate choice of an SRT approach even in frail patients with the worst OS prognoses (PS ≥ 2, RPA-3). Indeed compared to a WBRT, an SRT approach permitted us to avoid WBRT-induced toxicity, to reduce the number of fractions, and then to preserve the quality of life of frail patients [14,35].

The choice of SRT in our study led to an excellent LCR of 97.3% at 1 year and 96.4% at 2 years, and 77.1% of patients received only one or two subsequent SRTs. Furthermore, the median number of new BMs to be treated per patient throughout the time did not increase over time. The radiological radio-necrosis rate at 1 year was low at 2.1% (1.0–3.1), despite an initial and cumulative brain-PTV V12 (brain volume outside PTV receiving at least 12 Gy) of, respectively, 29.1 cm^3^ and 37.3 cm^3^, beyond the usual reported dose constraints [36,37]. We can also emphasize that the ultimate cumulative MBD was low (4.9 Gy). The tolerance of the treatment was excellent, with very few toxicities, consistent with other studies assessing the absence of quality-of-life or neurocognitive impairment after brain SRT [14,35].

Clinical experiences of ≥10 BM radiosurgery are mainly represented by Gamma-knife^®^ (GK) treatments [19,38,39,40,41]. In our study, all patients were treated with a Cyberknife^®^, which presents some advantages compared to the GK approach. A shorter treatment time and a frameless noninvasive positioning appear to be more convenient for patients.

Our study may also have some limitations. Firstly, there was no cognitive data collection; however, the low cumulative MBD values suggest that the healthy brain received a low dose of radiation that could not substantially impair cognitive functions [42,43]. Secondly, the number of patients is limited, although it represents the largest study in this population of patients with ≥10 BMs at initial BM presentation. Third, the reproducibility of these results could only be applied in respect to the preconditions mentioned above. However, we can underline that, during the same 5 years period, only 20 patients with ≥10 BMs at BM diagnosis could not benefit from an upfront SRT and received a WBRT at initial BM diagnosis.

Several prospective trials are ongoing to assess the place of SRT in cases of multiple BMs compared to WBRT (NCT04891471, NCT03775330, NCT04277403, NCT03550391, and NCT01592968) [44]. However, the further design of new clinical trials investigating this issue while proposing WBRT as a reference treatment may appear unethical regarding the efficacy and the low toxicity of this SRT approach in cases of ≥10 BMs at BM diagnosis.

In conclusion, providing that the aforementioned preconditions are met, an SRT should be proposed even for patients with ≥10 BMs at BM diagnosis.

## Figures and Tables

**Table 1:** Patient demographics and treatment characteristics at first treatment.

Demographics	N = 70 (%)
Median Age, years (range; min–max)	64.4 (24.8–86.1)
Sex	
Male	33 (47.1)
Female	37 (52.8)
Primary cancer	
NSCLC	36 (51.4)
Adenocarcinoma	33 (47.1)
Squamous cell	2 (2.9)
Giant cell	1 (1.4)
Melanoma	12 (17.0)
SCLC	8 (11.4)
Kidney	5 (7.1)
Breast	4 (5.8)
Other	5 (7.1)
Brain metastases	
Metachronous	41 (58.6)
Synchronous	29 (41.4)
ECOG PS	
0	18 (25.7)
1	38 (54.3)
≥2	14 (20)
RPA class	
1	11 (15.7)
2	45 (64.3)
3	14 (20)
Ds-GPA class	
2	2 (2.9)
3	40 (57.1)
4	28 (40.0)
Controlled primary	
Yes	18 (25.7)
No	52 (74.3)
Concomitant treatment	
None	19 (27.1)
Immunotherapy	21 (30.0)
Chemotherapy	10 (14.2)
Chemo-immunotherapy combination	6 (7.1)
Molecular targeted therapy	11 (15.7)
Initial treatment	(range; min–max)
Total number of metastases	N = 1174
Median number of metastases	14 (10–64)
Number of post-operative cavities	6 (0.5%)
Median PTV, cm^3^	7.0 (1.0–48.3)
Median GTV, cm^3^	3.0 (0.3–36.1)
Mean brain Dose, Gy	3.8 (1.3–8.9)
Median brain-PTV V12, cm^3^	29.1 (1.7–422)
Median treatment dose	20 (15–35)
Median number of fractions	1 (1–5)
Treatment regimen	
20 Gy in 1 fraction	775 (66.0%)
24 Gy in 1 fraction	348 (29.6%)
35 Gy in 5 fractions	18 (1.5%)
Other	33 (2.8%)

NSCLC: non-small-cell lung cancer; SCLC: small-cell lung cancer; ECOG performance status; RPA: recursive partitioning analysis; Ds-GPA: diagnosis-specific graded prognostic assessment; PTV: planning target volume, GTV: gross tumor volume. V12: volume receiving at least 12 Gy.

**Table 2:** Patient and lesions’ outcome.

Patient Outcome	N = 70 (%)
Median follow-up (months)	18.8 (3.6–55.1)
Overall survival	
At 1 year	62.3% (50.0–74.6)
At 2 years	48.7% (34.1–63.0)
Strategy success rate *	
At 1 year	77.8% (66.1–89.5)
At 2 years	68.8% (54.2–83.3)
Freedom from WBRT	
At 1 year	87.8% (78.8–96.8)
At 2 years	78.7% (66.0–91.5)
Cumulative number of brain treatments	
1	39 (55.7%)
2	15 (21.4%)
3	7 (10.0%)
≥4	9 (12.8%)
At least one radio-necrosis	
No	60 (85.7%)
Yes	10 (14.2%)
Grade 1	9 (12.8%)
Grade 2	1 (1.4%)
≥Grade 2 Toxicity	
No	67 (95.7%)
Yes	3 (4.3%)
Lesions outcome	(range)
Cumulative number of metastases	N = 1537
Local control rate	
At 1 year	97.3% (96.4–98.2)
At 2 years	96.4% (95.0–97.8)
Necrosis rate	
At 1 year	2.1% (1.0–3.1)
At 2 years	2.9% (1.5–4.3)
Median cumulative number of metastases	16 (10–105)
Median cumulative PTV, cm^3^	7.7 (0.9–54.7)
Cumulative mean brain dose, Gy	4.9 (1.3–11.0)
Cumulative number of brainstem metastases	25 (1.6%)
Median cumulative brain-PTV V12**, cm^3^	37.3 (1.7–529.3)

* Strategy success rate: proportion of patients without salvage whole-brain radiotherapy or death from neurological progression; V12**: volume receiving at least 12 Gy; BMs: brain metastases.

**Table 3:** Number of BMs and new BMs treated through time.

Treatment Sequence	Number of Treated Patients	Total Number of Treated Lesions	Median Number of Lesions per Patient (Min–Max Range)	Median Time from the Previous Sequence in Months (Min–Max Range)
N°1	70	1174	14 (10–64)	-
N°2	31	157	3(1–29)	4.3 (2.3–24.7)
N°3	16	100	4 (1–22)	8.2 (5.3–25.1)
N°4	9	69	3 (1–34)	12.0 (8.3–26.2)
N°5	4	17	4 (2–17)	22.1 (14.7–23.4)
N°6	3	21	8 (1–12)	17.4 (17.2–23.4)
N°7	1	1	1 (1–1)	12.3 (12.3–12.3)

**Table 4:** Prognosis factor for overall survival and strategy success rate at 1 year.

Variable (N)	Overall Survival (95% CI)	Log Rank *p*-Value/Cox Regression	Strategy Success Rate (95% CI)	Log Rank *p*-Value
Age		*p* = 0.82/NI		*p* = 0.63
<64 y-o (35)	65.3% (47.7–82.9)		76.0% (59.0–93.0)	
≥64 y-o (35)	59.5% (76.8–42.2)		80.0% (62.0–98.0)	
Sex		*p* = 0.023/NS		*p* = 0.35
Female (37)	69.3% (52.4–86.4)		80.0% (65.5–95.7)	
Male (33)	55.3% (37.7–72.9)		75.3% (58.1–92.5)	
ECOG PS		*p* = 0.002/NI **		*p* = 0.63
0–1 (56)	70.9% (58.0–83.8)		76.8% (64.1–89.5)	
≥2 (14)	25.4% (0–52.4)		87.5% (65.0–100)	
RPA class		*p* = 0.002/*p* = 0.0004		*p* = 0.63
1–2 (56)	70.9% (58.0–83.8)		76.8% (64.1–89.5)	
3 (14)	25.4% (0–52.4)		87.5% (65.0–100)	
Ds-GPA class		*p* = 0.24/NI		*p* = 0.56
≤3 (42)	69.7% (54.8–84.7)		80.3% (66.0–94.6)	
4 (28)	52.1% (32.2–72.0)		75.3% (56.4–94.3)	
Metastases		*p* = 0.33/NI		*p* = 0.16
Metachronous (41)	61.6% (45.5–77.3)		72.0% (55.4–88.6)	
Synchronous (29)	64.4% (45.4–83.4)		84.5% (68.0–100)	
Number of brain metastases		*p* = 0.50/NI		*p* = 0.31
<14 (35)	64.6% (46.8–82.2)		79.6% (63.3–95.9)	
≥14 (35)	60.4% (43.6–77.2)		76.1% (60.4–92.3)	
Tumor volume		*p* = 0.38/NI		*p* = 0.47
<PTV 7 cm^3^ (35)	70.4% (53.9–86.9)		72.5% (54.9–90.1)	
≥PTV 7 cm^3^ (35)	65.0% (52.5–77.6)		86.5% (72.2–100)	
Primary		*p* = 0.0375/NS		*p* = 0.34
SCLC (36)	46.7% (10.2–83.3)		80.0% (44,9–100)	
Other Primary (34)	64.3% (51.2–77.4)		77.2% (64.0–90.1)	
Controlled Primary		*p* = 0.08/NS		*p* = 0.88
Yes (18)	80.7% (64.7–96.6)		75.1% (53.9–96.3)	
No (52)	55.9% (41.2–70.6)		80.1% (66.8–93.0)	
Concomitant Treatment		*p* = 0.055/NS		*p* = 0.92
Molecular targeted therapy (11)	87.5% (64.5–100)		75.0% (45.0–100)	
Other treatment (59)	58.5% (45.2–71.8)		78.0% (65.0–91.0)	

ECOG PS: ECOG performance status; RPA: recursive partitioning analysis; Ds-GPA: diagnosis-specific graded prognostic assessment; PTV: planning target volume, SCLC: small-cell lung cancer; NI: not included; ** strongly correlated with RPA: not included; NS: non-significant.

## Data Availability

Dataset available on request from the authors.

## References

[1] Tsao M., Xu W., Sahgal A. (2012). A Meta-Analysis Evaluating Stereotactic Radiosurgery, Whole-Brain Radiotherapy, or Both for Patients Presenting with a Limited Number of Brain Metastases. Cancer.

[2] Schiappacasse L., Kinj R., Micheli R.D., Mederos N., Tuleasca C., Cossu G., Dunet V., Levivier M., Bourhis J., HOTTINGER A.F. (2022). Management of Brain Metastases in 2022. Rev. Medicale Suisse.

[3] Sacks P., Rahman M. (2020). Epidemiology of Brain Metastases. Neurosurg. Clin. N. Am..

[4] Lamba N., Wen P.Y., Aizer A.A. (2021). Epidemiology of Brain Metastases and Leptomeningeal Disease. Neuro-Oncology.

[5] Nabors L.B., Portnow J., Baehring J., Bhatia A., Bloch O., Brem S., Butowski N., Cannon D.M., Chao S., Chheda M.G. NCCN Guidelines Version 1.2023 Central Nervous System Cancers Continue NCCN Guidelines Panel Disclosures 2023. https://www.nccn.org/guidelines/guidelines-detail?category=1&id=1425.

[6] Rhun E.L., Guckenberger M., Smits M., Dummer R., Bachelot T., Sahm F., Galldiks N., de Azambuja E., Berghoff A.S., Metellus P. (2021). EANO–ESMO Clinical Practice Guidelines for Diagnosis, Treatment and Follow-up of Patients with Brain Metastasis from Solid Tumours. Ann. Oncol..

[7] Moravan M.J., Fecci P.E., Anders C.K., Clarke J.M., Salama A.K.S., Adamson J.D., Floyd S.R., Torok J.A., Salama J.K., Sampson J.H. (2020). Current Multidisciplinary Management of Brain Metastases. Cancer.

[8] Suh J.H., Kotecha R., Chao S.T., Ahluwalia M.S., Sahgal A., Chang E.L. (2020). Current Approaches to the Management of Brain Metastases. Nat. Rev. Clin. Oncol..

[9] Ali S., Górska Z., Duchnowska R., Jassem J. (2021). Molecular Profiles of Brain Metastases: A Focus on Heterogeneity. Cancers.

[10] Antoni D., Clavier J.B., Pop M., Schumacher C., Lefebvre F., Noël G. (2013). Institutional, Retrospective Analysis of 777 Patients with Brain Metastases: Treatment Outcomes and Diagnosis-Specific Prognostic Factors. Int. J. Radiat. Oncol. Biol. Phys..

[11] Windsor A.A., Koh E.S., Allen S., Gabriel G.S., Yeo A.E.T., Allison R., van der Linden Y.M., Barton M.B. (2013). Poor Outcomes after Whole Brain Radiotherapy in Patients with Brain Metastases: Results from an International Multicentre Cohort Study. Clin. Oncol..

[12] Minniti G., Clarke E., Lanzetta G., Osti M.F., Trasimeni G., Bozzao A., Romano A., Enrici R.M. (2011). Stereotactic Radiosurgery for Brain Metastases: Analysis of Outcome and Risk of Brain Radionecrosis. Radiat. Oncol..

[13] Van Grinsven E.E., Nagtegaal S.H.J., Verhoeff J.J.C., Van Zandvoort M.J.E. (2021). The Impact of Stereotactic or Whole Brain Radiotherapy on Neurocognitive Functioning in Adult Patients with Brain Metastases: A Systematic Review and Meta-Analysis. Oncol. Res. Treat..

[14] Schimmel W.C.M., Gehring K., Eekers D.B.P., Hanssens P.E.J., Sitskoorn M.M. (2018). Cognitive Effects of Stereotactic Radiosurgery in Adult Patients with Brain Metastases: A Systematic Review. Adv. Radiat. Oncol..

[15] Latorzeff I., Antoni D., Josset S., Noël G., Tallet-Richard A. (2022). Radiation Therapy for Brain Metastases. Cancer/Radiothérapie.

[16] Yamamoto M., Serizawa T., Shuto T., Akabane A., Higuchi Y., Kawagishi J., Yamanaka K., Sato Y., Jokura H., Yomo S. (2014). Stereotactic Radiosurgery for Patients with Multiple Brain Metastases (JLGK0901): A Multi-Institutional Prospective Observational Study. Lancet Oncol..

[17] Rusthoven C.G., Yamamoto M., Bernhardt D., Smith D.E., Gao D., Serizawa T., Yomo S., Aiyama H., Higuchi Y., Shuto T. (2020). Evaluation of First-Line Radiosurgery vs. Whole-Brain Radiotherapy for Small Cell Lung Cancer Brain Metastases: The FIRE-SCLC Cohort Study. JAMA Oncol..

[18] Gondi V., Bauman G., Bradfield L., Burri S.H., Cabrera A.R., Cunningham D.A., Eaton B.R., Hattangadi-Gluth J.A., Kim M.M., Kotecha R. (2022). Radiation Therapy for Brain Metastases: An ASTRO Clinical Practice Guideline. Pract. Radiat. Oncol..

[19] Yamamoto M., Serizawa T., Sato Y., Higuchi Y., Kasuya H. (2021). Stereotactic Radiosurgery Results for Patients with 5–10 versus 11–20 Brain Metastases: A Retrospective Cohort Study Combining 2 Databases Totaling 2319 Patients. World Neurosurg..

[20] Wei Z., Luy D.D., Jose S., Deng H., Yavan S., Worrell S., Belkhir J.R., Tang L.W., Lunsford L.D. (2023). Single-Session Gamma Knife Radiosurgery for Patients with 20 or More Brain Metastases. Neurosurgery.

[21] Gaspar L., Scott C., Rotman M., Asbell S., Phillips T., Wasserman T., Kenna W.M.G., Byhardt R. (1997). Recursive Partitioning Analysis (Rpa) Of Prognostic Factors. Three Radiation Therapy Oncology Group (Rtog) Brain Metastases Trials.

[22] Nieder C., Andratschke N.H., Geinitz H., Grosu A.L. (2012). Diagnosis-Specific Graded Prognostic Assessment Score Is Valid in Patients with Brain Metastases Treated in Routine Clinical Practice in Two European Countries. Med. Sci. Monit. Int. Med. J. Exp. Clin. Res..

[23] Sperduto P.W., Berkey B., Gaspar L.E., Mehta M., Curran W. (2008). A New Prognostic Index and Comparison to Three Other Indices for Patients with Brain Metastases: An Analysis of 1960 Patients in the RTOG Database. Int. J. Radiat. Oncol. Biol. Phys..

[24] Cancer Institute N. (2009). Common Terminology Criteria for Adverse Events (CTCAE) Common Terminology Criteria for Adverse Events v4.0 (CTCAE). Clin. Cancer Res..

[25] Kaufmann T.J., Smits M., Boxerman J., Huang R., Barboriak D.P., Weller M., Chung C., Tsien C., Brown P.D., Shankar L. (2020). Consensus Recommendations for a Standardized Brain Tumor Imaging Protocol for Clinical Trials in Brain Metastases. Neuro-Oncol..

[26] Kano H., Kondziolka D., Lobato-Polo J., Zorro O., Flickinger J.C., Lunsford L.D. (2010). T1/T2 Matching to Differentiate Tumor Growth from Radiation Effects after Stereotactic Radiosurgery. Neurosurgery.

[27] Lin N., Lin N.U., Lee E.Q., Aoyama H., Barani I.J., Barboriak D.P., Baumert B.G., Bendszus M., Brown P.D., Camidge R. (2015). Response Assessment Criteria for Brain Metastases: Proposal from the RANO Group. Lancet Oncol..

[28] Kishore J., Goel M., Khanna P. (2010). Understanding Survival Analysis: Kaplan-Meier Estimate. Int. J. Ayurveda Res..

[29] Bland J.M., Altman D.G. (2004). Statistics Notes The Logrank Test. BMJ.

[30] Dessai S., Patil V. (2019). Testing and Interpreting Assumptions of COX Regression Analysis. Cancer Res. Stat. Treat..

[31] Hughes R.T., Masters A.H., McTyre E.R., Farris M.K., Chung C., Page B.R., Kleinberg L.R., Hepel J., Contessa J.N., Chiang V. (2019). Initial SRS for Patients With 5 to 15 Brain Metastases: Results of a Multi-Institutional Experience. Int. J. Radiat. Oncol. Biol. Phys..

[32] Ali M.A., Hirshman B.R., Wilson B., Carroll K.T., Proudfoot J.A., Goetsch S.J., Alksne J.F., Ott K., Aiyama H., Nagano O. (2017). Survival Patterns of 5750 Stereotactic Radiosurgery–Treated Patients with Brain Metastasis as a Function of the Number of Lesions. World Neurosurg..

[33] Tatineni V., O’Shea P.J., Saxena S., Khosla A.A., Ozair A., Kotecha R.R., Jia X., Rauf Y., Murphy E.S., Chao S.T. (2023). Combination of EGFR-Directed Tyrosine Kinase Inhibitors (EGFR-TKI) with Radiotherapy in Brain Metastases from Non-Small Cell Lung Cancer: A 2010–2019 Retrospective Cohort Study. Cancers.

[34] Tozuka T., Noro R., Mizutani H., Kurimoto F., Hakozaki T., Hisakane K., Naito T., Takahashi S., Taniuchi N., Yajima C. (2024). Osimertinib plus Local Treatment for Brain Metastases versus Osimertinib Alone in Patients with EGFR-Mutant Non-Small Cell Lung Cancer. Lung Cancer.

[35] Verhaak E., Gehring K., Hanssens P.E.J., Aaronson N.K., Sitskoorn M.M. (2020). Health-Related Quality of Life in Adult Patients with Brain Metastases after Stereotactic Radiosurgery: A Systematic, Narrative Review. Support. Care Cancer.

[36] Korytko T., Radivoyevitch T., Colussi V., Wessels B.W., Pillai K., Maciunas R.J., Einstein D.B. (2006). 12 Gy Gamma Knife Radiosurgical Volume Is a Predictor for Radiation Necrosis in Non-AVM Intracranial Tumors. Int. J. Radiat. Oncol. Biol. Phys..

[37] Flickinger J.C., Kondziolka D., Lunsford L.D., Pollock B.E., Yamamoto M., Gorman D.A., Schomberg P.J., Sneed P., Larson D., Smith V. (1999). PII S0360-3016(98)00518-5 clinical investigation central nervous system a multi-institutional analysis of complication outcomes after arteriovenous malformation radiosurgery. Int. J. Radiat. Oncol. Biol. Phys..

[38] Grandhi R., Kondziolka D., Panczykowski D., Monaco E.A., Kano H., Niranjan A., Flickinger J.C., Lunsford L.D. (2012). Stereotactic Radiosurgery Using the Leksell Gamma Knife Perfexion Unit in the Management of Patients with 10 or More Brain Metastases: Clinical Article. J. Neurosurg..

[39] Raldow A.C., Chiang V.L., Knisely J.P., Yu J.B. (2013). Survival and Intracranial Control of Patients with 5 or More Brain Metastases Treated with Gamma Knife Stereotactic Radiosurgery. Am. J. Clin. Oncol. Cancer Clin. Trials.

[40] Rava P., Leonard K., Sioshansi S., Curran B., Wazer D.E., Cosgrove G.R., Norén G., Hepel J.T. (2013). Survival among Patients with 10 or More Brain Metastases Treated with Stereotactic Radiosurgery. J. Neurosurg..

[41] Chang W.S., Kim H.Y., Chang J.W., Park Y.G., Chang J.H. (2010). Analysis of Radiosurgical Results in Patients with Brain Metastases According to the Number of Brain Lesions: Is Stereotactic Radiosurgery Effective for Multiple Brain Metastases?. J. Neurosurg..

[42] Zhang L., Sun R., Tian Y. (2011). Dose-Response Relationship for Radiation-Induced Cognitive Impairment. Int. J. Radiat. Oncol. Biol. Phys..

[43] Yamamoto M., Serizawa T., Higuchi Y., Sato Y., Kawagishi J., Yamanaka K., Shuto T., Akabane A., Jokura H., Yomo S. (2017). A Multi-Institutional Prospective Observational Study of Stereotactic Radiosurgery for Patients With Multiple Brain Metastases (JLGK0901 Study Update): Irradiation-Related Complications and Long-Term Maintenance of Mini-Mental State Examination Scores. Int. J. Radiat. Oncol. Biol. Phys..

[44] Tini P., Marampon F., Giraffa M., Bucelli S., Niyazi M., Belka C., Minniti G. (2023). Current Status and Perspectives of Interventional Clinical Trials for Brain Metastases: Analysis of ClinicalTrials.Gov. Radiat. Oncol..

